# Loss of giant obscurins alters breast epithelial cell mechanosensing of matrix stiffness

**DOI:** 10.18632/oncotarget.10997

**Published:** 2016-08-01

**Authors:** Kimberly M. Stroka, Bin Sheng Wong, Marey Shriver, Jude M. Phillip, Denis Wirtz, Aikaterini Kontrogianni-Konstantopoulos, Konstantinos Konstantopoulos

**Affiliations:** ^1^ Fischell Department of Bioengineering, University of Maryland, College Park, MD, 20742, USA; ^2^ Johns Hopkins Institute for NanoBioTechnology, The Johns Hopkins University, Baltimore, MD, 21218, USA; ^3^ Johns Hopkins Physical Sciences-Oncology Center, The Johns Hopkins University, Baltimore, MD, 21218, USA; ^4^ Department of Chemical and Biomolecular Engineering, The Johns Hopkins University, Baltimore, MD, 21218, USA; ^5^ University of Maryland School of Medicine, Department of Biochemistry and Molecular Biology, Baltimore, MD, 21201, USA; ^6^ University of Maryland School of Medicine, Marlene and Stewart Greenebaum National Cancer Institute Cancer Center, Baltimore, MD, 21201, USA

**Keywords:** matrix stiffness, mechanosensitivity, cell migration, obscurin, RhoA

## Abstract

Obscurins are a family of RhoGEF-containing proteins with tumor and metastasis suppressing roles in breast epithelium. Downregulation of giant obscurins in normal breast epithelial cells leads to reduced levels of active RhoA and of its downstream effectors. Herein, we elucidate how depletion of giant obscurins affects the response of breast epithelial cells to changes in the mechanical properties of the microenvironment. We find that knockdown of obscurins increases cell morphodynamics, migration speed, and diffusivity on polyacrylamide gels of ≥ 1 kPa, presumably by decreasing focal adhesion area and density as well as cell traction forces. Depletion of obscurins also increases cell mechanosensitivity on soft (0.4–4 kPa) surfaces. Similar to downregulation of obscurins, pharmacological inhibition of Rho kinase in breast epithelial cells increases migration and morphodynamics, suggesting that suppression of Rho kinase activity following obscurin knockdown can account for alterations in morphodynamics and migration. In contrast, inhibition of myosin light chain kinase reduces morphodynamics and migration, suggesting that temporal changes in cell shape are required for efficient migration. Collectively, downregulation of giant obscurins facilitates cell migration through heterogeneous microenvironments of varying stiffness by altering cell mechanobiology.

## INTRODUCTION

As tumor cells spread during metastasis, they must navigate through complex physical and biochemical microenvironments [1]. In particular, the stiffness of the cellular microenvironment not only varies throughout different tissues *in vivo* [2], but also influences cell migration via alterations in cell signaling pathways [3–7]. Furthermore, tissue stiffness increases in many cancers [2, 8], likely due to the alterations in extracellular matrix (ECM) composition during tumor growth [9]. Metastasizing tumor cells must therefore possess the ability to migrate along substrates and through matrices of a wide range of stiffnesses. Currently, it is mostly unknown what genetic changes allow cells to alter their mechanobiology and respond to these varying mechanical properties of the microenvironment during metastasis. Recent findings from our labs have implicated giant obscurins in breast cancer progression and metastasis [10–12], and here we explore their role in cell mechanobiology and mechanosensing.

Obscurins, encoded by the single *OBSCN* gene, are a family of giant cytoskeletal proteins that have been mostly studied in the context of striated muscle cell organization and function [13–21]. The human *OBSCN* gene spans 150 kb on chromosome 1q42 and gives rise to at least four isoforms via alternative splicing [20, 22]. Giant obscurins A (˜720 kDa) and B (˜870 kDa) contain multiple signaling and adhesion domains arranged in tandem [23], including a Rho-guanine nucleotide exchange factor (Rho-GEF). *OBSCN* was identified as one of 189 “candidate cancer genes” in breast and colorectal cancers due to its high mutational frequency [24]. Of those 189 genes, only *OBSCN* and *TP53* were common to both breast and colorectal cancers. Consistent with these observations, we have demonstrated that giant obscurins are abundantly expressed in non-tumorigenic breast epithelial cell lines and normal breast tissue, but are nearly absent from breast cancer cell lines and advanced grade (grade-2 and higher) human breast cancer biopsies [10, 25]. Depletion of giant obscurins from non-tumorigenic MCF10A breast epithelial cells promotes apoptotic resistance [10], disrupts adherens junctions, increases cell migration and invasion *in vitro*, and potentiates tumorigenicity and metastasis *in vivo* [12]. These alterations are attributed to the critical role of obscurins in cell cytoskeletal organization and dynamics [11, 12, 26]. The cell cytoskeleton is largely regulated by the family of RhoGTPases, including RhoA, which has been implicated in the regulation of cell mechanosensitivity in microenvironments of varying stiffness [27, 28]. Rho GTPases, including RhoA, regulate stress fibers and focal adhesions [29], two structures whose assembly is tightly controlled by matrix stiffness. Stiffer substrates reinforce integrin-cytoskeletal connections at focal adhesions, possibly via a molecular clutch mechanism [30], leading to enhanced stress fiber formation and elevated RhoA activity.

RhoA is activated via the obscurin RhoGEF domain [11]. Depletion of giant obscurins from MCF10A cells (both attached and suspended) significantly reduces RhoA activity and thus phosphorylation of RhoA downstream effectors, including myosin light chain phosphatase, myosin light chain (MLC), lim kinase, and cofilin [11]. We therefore hypothesize that depletion of giant obscurins from MCF-10A breast epithelial cells alters cell mechanosensitivity via the RhoA pathway. We herein delineate the role of obscurins in cell mechanobiology and mechanosensing of matrix stiffness. We demonstrate that loss of giant obscurins alters cell morphology, increases morphodynamics and mechanosensitivity, and affects focal adhesion morphology and traction forces. Together, our results indicate that loss of giant obscurins facilitates cell migration through heterogeneous microenvironments of varying stiffness by altering cell mechanobiology via RhoA-mediated effects.

## RESULTS

### Loss of giant obscurins alters breast epithelial cell morphology and morphodynamics

**Figure 1:**
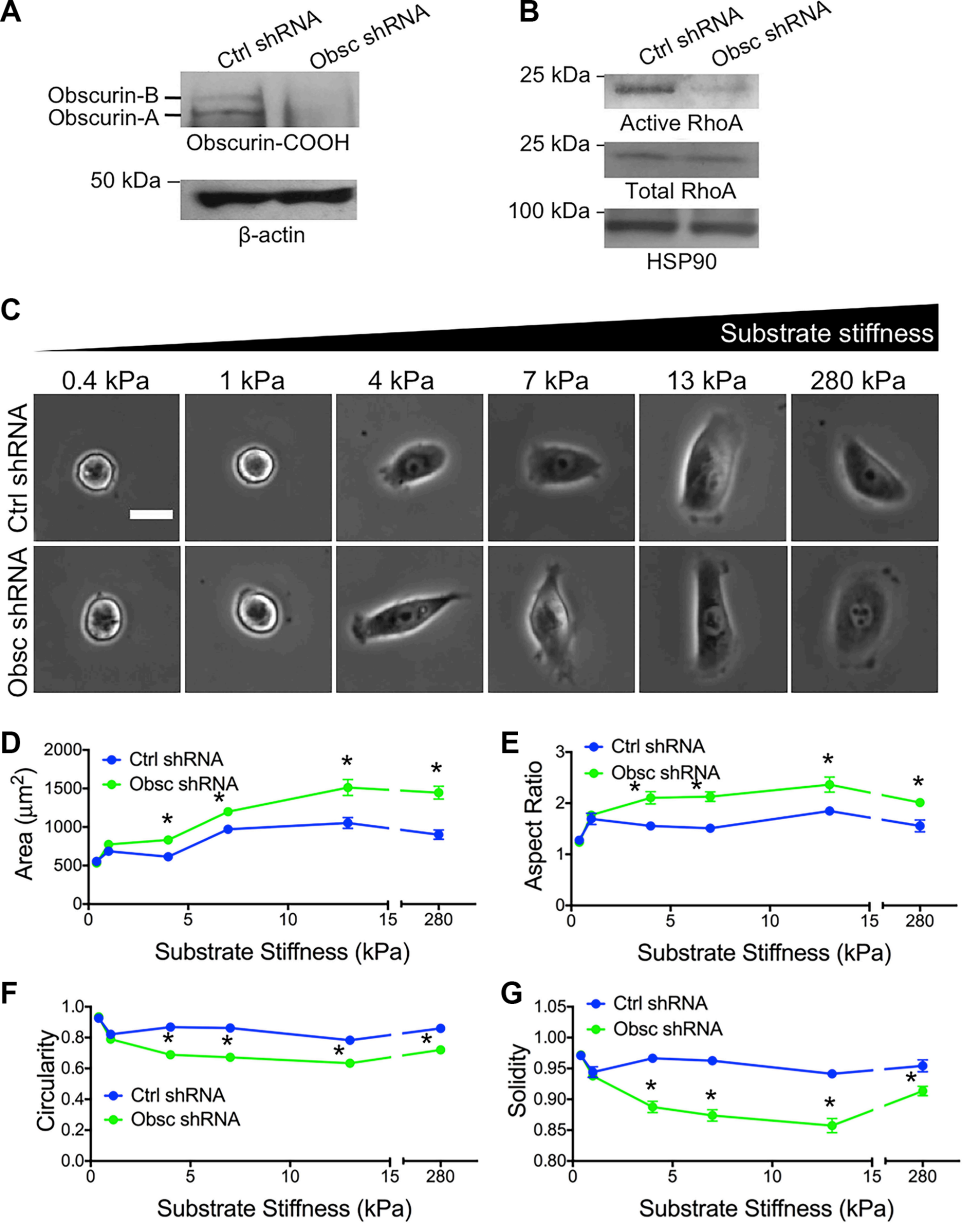
Morphology of MCF10A breast epithelial cells expressing scramble control shRNA (Ctrl shRNA) and obscurin shRNA (Obsc shRNA) on substrates of varying stiffness (**A**) Western blots of Obscurin-COOH expression in MCF10A with Ctrl shRNA and Obsc shRNA. (**B**) Total and active RhoA in MCF10A with Ctrl shRNA and Obsc shRNA. (**C**) Representative phase contrast images of MCF10A Ctrl shRNA and Obsc shRNA on fibronectin-coated polyacrylamide gels of varying stiffness, ranging from 0.4 to 280 kPa, after 20 h of spreading. Scale bar represents 25 μm and applies to all images. Comparison of the various morphology parameters, including spreading area (**D**), aspect ratio (**E**), circularity (**F**) and solidity (**G**), between MCF10A Ctrl shRNA and Obsc shRNA across different substrate stiffness. *p < 0.05 relative to control of same stiffness using a paired Student's *t*-test.

Cell morphology is a critical parameter when evaluating cellular responses to matrix mechanical properties. Numerous cell types have shown differential spreading behavior depending on substrate stiffness [3, 31]. We therefore aimed to evaluate the role of giant obscurins in breast epithelial cell morphological response to matrix mechanics. As we previously reported, MCF10A cells stably expressing obscurin shRNA, but not scramble shRNA, display robust down-regulation of giant obscurins, as determined by Western Blot analysis (Figure 1A), and decreased levels of active RhoA (Figure 1B) [11]. MCF10A cells expressing scramble control or obscurin shRNA were plated onto fibronectin-coated polyacrylamide gels of varying stiffness and allowed to attach and spread for approximately 20 h. Cell morphology parameters, including spreading area, aspect ratio, circularity, and solidity (as defined in Materials and Methods section) were measured using phase contrast images. These parameters define the area of the cell projected on a 2D surface (spreading area), elongation (aspect ratio), roundness (circularity), and protrusivity (solidity) of the cell. It should be noted that a circularity and solidity value of 1 defines a completely round cell. As the cell becomes more protrusive, solidity decreases (down to a minimum of 0). Meanwhile, as the cell elongates, circularity decreases (down to a minimum of 0), while aspect ratio increases.

In both scramble control and obscurin knockdown cells, spreading area increased with substrate stiffness, though to a greater degree in the case of obscurin knockdown cells (Figure 1C–1D) and reached a maximum level at 13 kPa. On substrates with stiffness of 1 kPa or greater, the aspect ratio, circularity, and solidity were mostly stiffness-independent (Figure 1E–1G). It is noteworthy that there was no significant difference in any cell morphology parameters between control and obscurin knockdown cells on 0.4 kPa where cells were mostly rounded and unspread (and remained unattached, as cells underwent large displacements when gently moving the well plate back and forth), or on 1 kPa where cells just began to spread and elongate (Figure 1). On substrates of 4 kPa or higher, obscurin knockdown cells had a significantly higher spreading area and aspect ratio (Figure 1D–1E), as well as significantly lower circularity and solidity (Figure 1F–1G), indicative of increased cell protrusivity. Taken together, these results indicate that obscurin downregulation has a significant effect on cell morphology on substrates 4 kPa and higher, and that spreading area is the morphology parameter most dependent on substrate stiffness for both control and obscurin knockdown cells.

**Figure 2:**
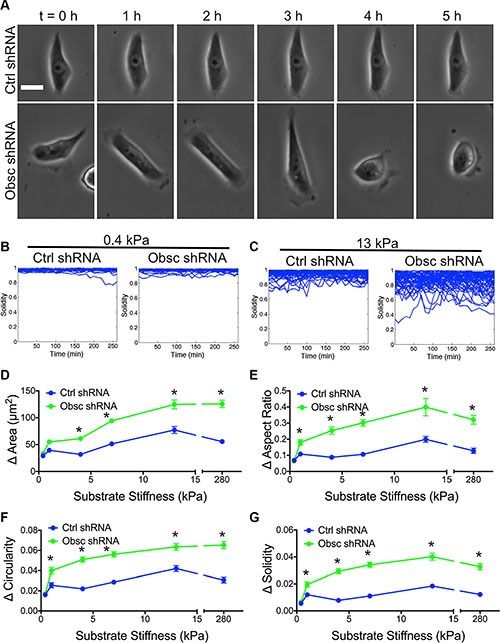
Morphodynamics of MCF10A Ctrl shRNA and Obsc shRNA on substrates of varying stiffness (**A**) Representative phase contrast images of MCF10A Ctrl shRNA and Obsc shRNA on 13 kPa polyacrylamide gels illustrating changes of cell morphology over time. The time interval between each image is 1 h. Scale bar represents 25 μm and applies to all images. Graphs showing the raw solidity values of MCF10A Ctrl shRNA and Obsc shRNA over 5 h on 0.4 kPa (**B**) and 13 kPa (**C**) polyacrylamide gels indicate that cells only alter shape dynamically on stiffer substrates (> 0.4 kPa). Also shown is the comparison of the magnitude of the change in morphology parameters, including spreading area (**D**), aspect ratio (**E**), circularity (**F**) and solidity (**G**), between MCF10A Ctrl shRNA and Obsc shRNA across different substrate stiffness. *p < 0.05 relative to control of same stiffness. Full ANOVA and Games-Howell post-hoc test results between stiffnesses are listed in Table S1.

We have previously reported that obscurin knockdown increases actin dynamics and cell migration velocity [12]. As cells migrate on two-dimensional (2D) surfaces, they dynamically change shape over time [32]. We therefore hypothesize that obscurin knockdown relative to scramble control cells exhibit increased morphodynamics, which is defined as the change in cell shape over time. To test this hypothesis, images were captured as cells migrated on different substrates over time (Figure 2A). The change in morphology parameters between two consecutive time points (i.e., every 10 min) was quantified. On 0.4 kPa, essentially no morphodynamics was noted in either the scramble control or obscurin knockdown cells, where, for example, plots of solidity versus time were mostly horizontal lines at values close to 1 (Figure 2B). In marked contrast, on 13 kPa, solidity displayed marked variations over time with values ranging from approximately 0.3 to 1 (Figure 2C). Changes in morphology parameters, such as spreading area, aspect ratio, circularity and solidity, increased as substrate stiffness increased from 0.4 to 13 kPa. In general, there was a striking increase in cell morphodynamics in obscurin knockdown relative to scramble control cells on substrates with stiffness of 1 kPa and higher (Figure 2D–2G; Table S1). These results indicate that loss of giant obscurins significantly increases cell morphodynamics on substrates 1 kPa and stiffer, whereas it has little effect on very soft surfaces of 0.4 kPa.

### Loss of giant obscurins alters breast epithelial cell migration and mechanosensitivity

**Figure 3:**
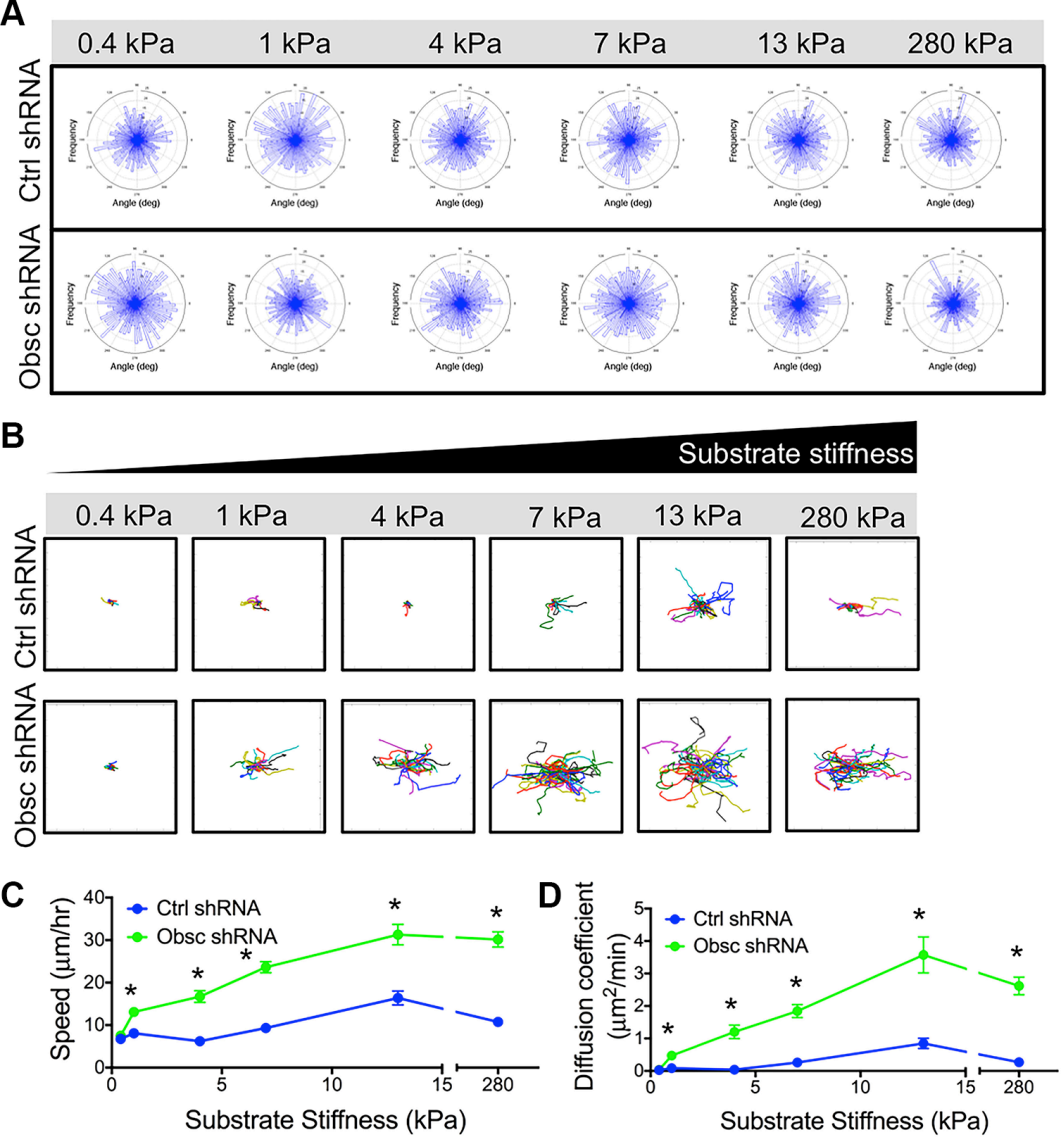
2D migration behaviors of MCF10A Ctrl shRNA and Obsc shRNA on substrates of varying stiffness (**A**) Turning angles of MCF10A Ctrl shRNA and Obsc shRNA migrating on polyacrylamide gels of varying stiffness showing a lack of preferred turning angles, indicating that cells are undergoing chemokinesis and are migrating randomly on the substrates. Individual cell trajectories (**B**), speed (**C**) and diffusion coefficient (**D**) of MCF10A Ctrl shRNA and Obsc shRNA migrating on 2D polyacrylamide gels of different stiffness. *p < 0.05 relative to control of same stiffness. Full ANOVA and Games-Howell post-hoc test results between stiffnesses are listed in Table S2.

Loss of giant obscurins increases cell migration velocity in response to a chemoattractant gradient in both wide and narrow microfluidic channels [12]. Here, we aimed to evaluate whether obscurins play a role in chemokinesis (random migration in the absence of a chemoattractant gradient) on the fibronectin-coated polyacrylamide gels. Indeed, scramble control and obscurin knockdown cells on all substrates undergo chemokinesis, as demonstrated by the lack of a preferred turning angle during migration across the substrates, resulting in similar radial histograms for all conditions (Figure 3A). Overall, both control and obscurin knockdown cells were more migratory as substrate stiffness increased from 0.4 to 13 kPa (Figure 3B–3C; Table S2). On softer substrates ranging from 0.4 to 4 kPa, there was no apparent increase in scramble control cell migration, as evidenced by the individual cell trajectories (Figure 3B), the quantified migration speed (Figure 3C; Table S2) and diffusion coefficient (Figure 3D; Table S2). In the same soft stiffness range, both the speed and the diffusion coefficient of obscurin knockdown cells increased significantly (Figure 3B–3D; Table S2), indicating their increased mechanosensitivity. For both control and obscurin knockdown cells, speed and diffusion coefficients reached peak values at 13 kPa (Figure 3C–3D). On all substrates with stiffness of 1 kPa and higher, obscurin knockdown cells were significantly faster (Figure 3C; Table S2) with higher diffusion coefficients (Figure 3D; Table S2) than scramble control cells.

### ROCK inhibition increases cell morphodynamics and mechanosensitivity in scramble control cells

**Figure 4:**
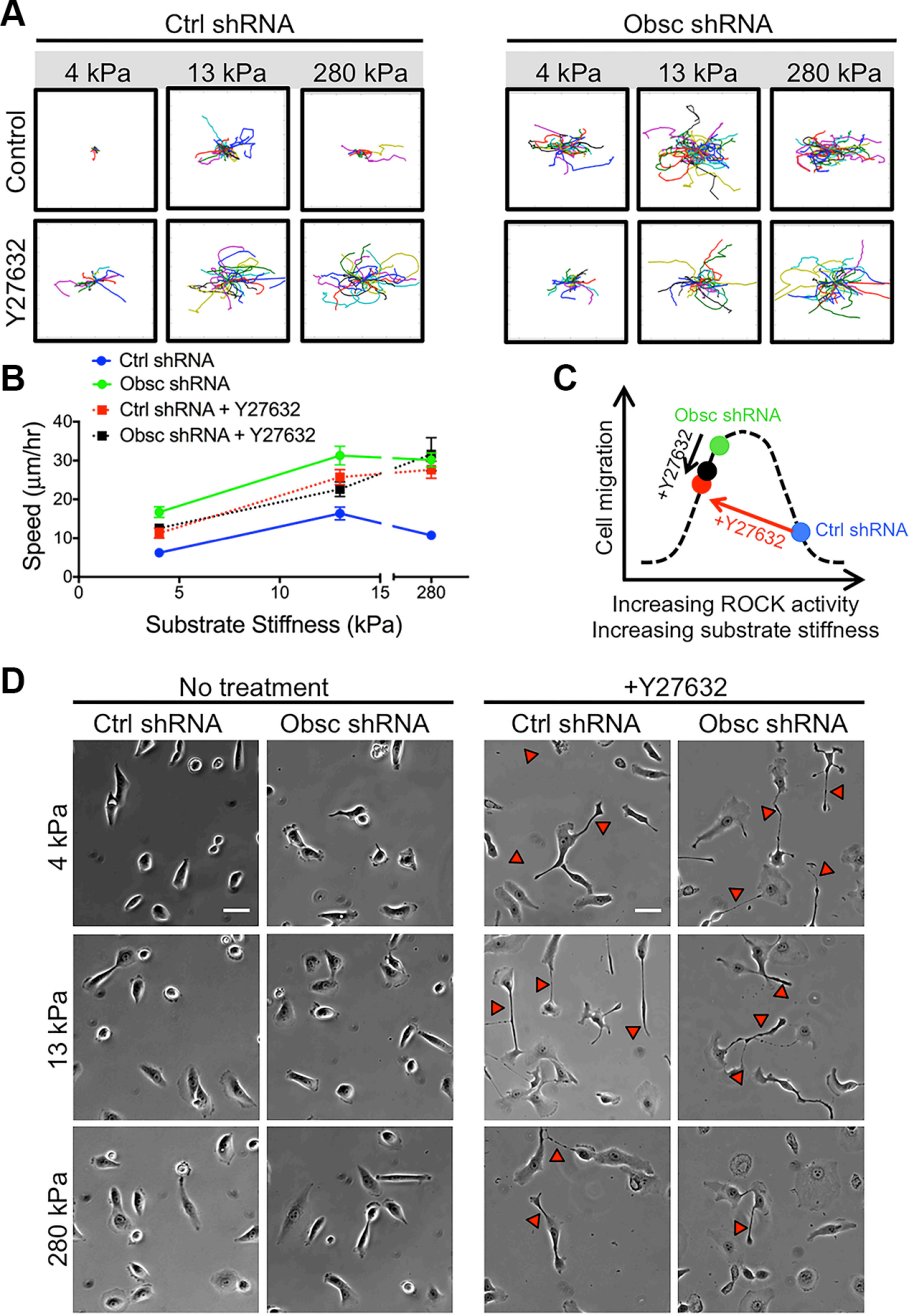
2D migration behaviors of MCF10A Ctrl shRNA and Obsc shRNA after ROCK inhibition with Y27632 on substrates of varying stiffness (**A**) Individual cell trajectories of MCF10A Ctrl shRNA and Obsc shRNA migrating on 4, 13 and 280 kPa polyacrylamide gels after treatment with 30 μM Y27632. (**B**) Speed comparison between untreated and Y27632-treated MCF10A Ctrl shRNA and Obsc shRNA across different substrate stiffness. (**C**) Schematic illustrating our hypothesis that there exists a biphasic relationship between cell contractility (mediated by ROCK activity or substrate stiffness) and cell migration, as well as the proposed effects of Y27632 on scramble control and obscurin knockdown cells. (**D**) Phase contrast images are shown for no treatment and 1 h after treatment of MCF10A Ctrl shRNA and Obsc shRNA cells with the ROCK inhibitor Y27632. Red arrows correspond to long tails indicating failure of cells to fully retract their rear during migration. Scale bar in panel (D) corresponds to 50 μm and applies to all images. Full ANOVA and Games-Howell post-hoc test results are listed in Table S3.

We have previously shown that knockdown of giant obscurins from normal breast epithelial cells significantly reduces RhoA activity (Figure 1B) [11], resulting in significantly decreased levels of phosphorylation (and thus activation) of several downstream effectors of RhoA [11]. Rho kinase (ROCK) is one downstream target of RhoA. To determine whether the increased migration of obscurin knockdown cells is at least partly due to decreased ROCK activity, scramble control cells were treated with the ROCK inhibitor Y27632 on polyacrylamide gels of prescribed stiffness ranging from 4 to 280 kPa where morphological differences were noted between control and obscurin knockdown cells. Y27632 significantly increased migration of scramble control cells on all substrates (Figure 4A–4B; Table S3), similar to the effects of obscurin knockdown. Interestingly, in obscurin knockdown cells, Y27632 failed to significantly affect cell migration in all stiffness examined (4, 13 or 280 kPa) in this work (Figure 4A–4B; Table S3). These data suggest that there exists an optimal cell contractility level necessary for maximal migration, which can be achieved either through optimal internal ROCK activity or through external mechanical cues such as matrix stiffness (Figure 4C). Indeed, the largest increase in scramble control cell migration speed following Y27632 treatment was observed on the stiffest (280 kPa) gel (Figure 4B), where cell contractility was likely the highest (Figure 4C).

**Figure 5:**
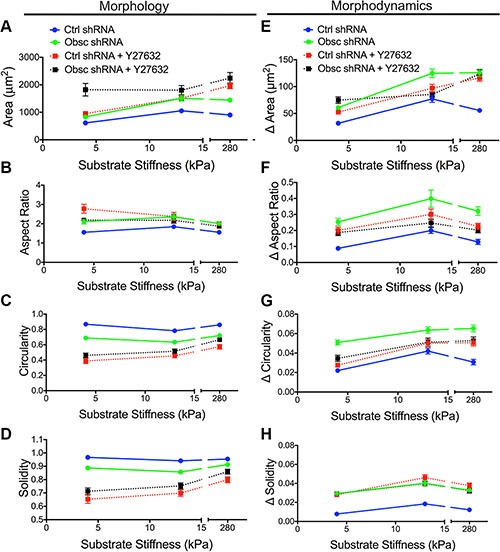
Morphology and morphodynamics of MCF10A Ctrl shRNA and Obsc shRNA cells following ROCK inhibition with Y27632 Comparison of the various morphology parameters, including spreading area (**A**), aspect ratio (**B**), circularity (**C**) and solidity (**D**), between untreated and Y27632-treated MCF10A Ctrl shRNA and Obsc shRNA cells across different substrate stiffness. Comparison of the change in morphology parameters (morphodynamics), including spreading area (**E**), aspect ratio (**F**), circularity (**G**) and solidity (**H**), between untreated and Y27632-treated MCF10A Ctrl shRNA and Obsc shRNA across different substrate stiffness. Full ANOVA and Games-Howell post-hoc test results are listed in Tables S4 and S5.

Phase contrast images were used to characterize the morphology of scramble control and obscurin knockdown cells upon treatment with Y27632 (Figure 4D). In scramble control cells, inhibition of ROCK via Y27632 increased cell spreading area and aspect ratio but decreased cell circularity and solidity on all substrates in the 4-280 kPa range, similar to the effects of obscurin knockdown (Figure 5A–5D; Table S4). In obscurin knockdown cells, treatment with Y27632 modestly increased cell area and decreased cell circularity and solidity (Figure 5A–5D; Table S4). These changes in cell morphology were expected, since inhibition of ROCK prevents cells from applying traction and detaching at the rear during migration (Figure 4D), which leads to larger cell areas and increased protrusivity.

Scramble control cells treated with Y27632 demonstrated increased morphodynamics (changes in area, aspect ratio, circularity, and solidity) on most substrates, similar to the effects of obscurin knockdown (Figure 5E–5H; Table S5). These results suggest that the accompanying decrease in RhoA activity upon obscurin knockdown may be responsible for the increased morphodynamics in migrating breast epithelial cells. Meanwhile, obscurin knockdown cells treated with Y27632 displayed little change in cell morphodynamics, with statistically significant changes only on some substrates (Figure 5E–5H; Table S5), indicating that further inhibiting ROCK activity in obscurin-depleted cells whose ROCK is likely already low, reduces cell morphodynamics on those substrates, along with a reduction in migratory potential on substrates softer than 280 kPa.

### MLCK inhibition decreases cell morphodynamics and migration in obscurin-depleted cells

**Figure 6:**
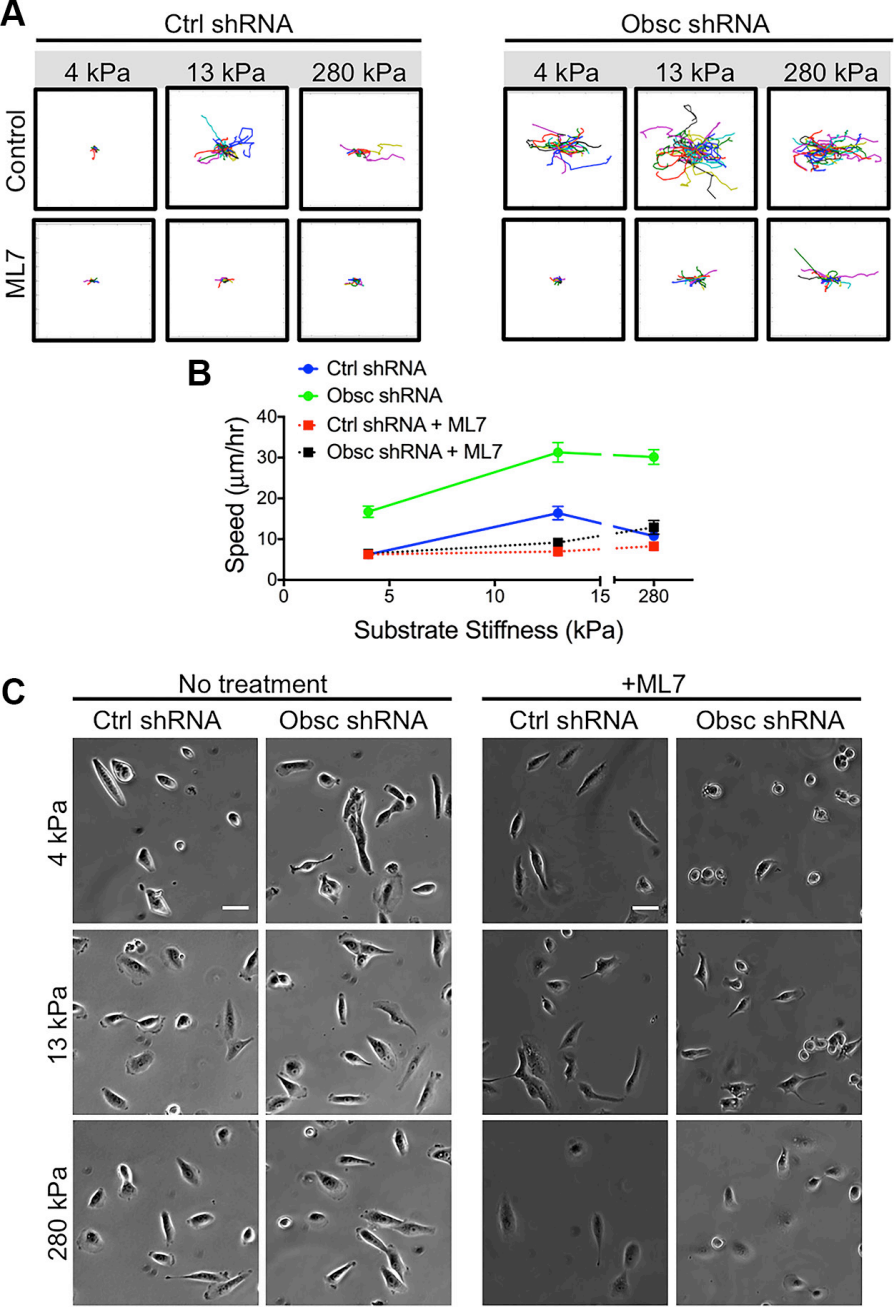
2D migration behaviors of MCF10A Ctrl shRNA and Obsc shRNA after MLCK inhibition with ML7 on substrates of varying stiffness (**A**) Individual cell trajectories of MCF10A Ctrl shRNA and Obsc shRNA migrating on 4, 13 and 280 kPa polyacrylamide gels after treatment with 25 μM ML7. (**B**) Speed comparison between untreated and ML7-treated MCF10A Ctrl shRNA and Obsc shRNA across different substrate stiffness. (**C**) Phase contrast images are shown for no treatment and 1 h after treatment of MCF10A Ctrl shRNA and Obsc shRNA cells with the MLCK inhibitor ML7. Scale bar in panel (C) corresponds to 50 μm and applies to all images. Full ANOVA and Games-Howell post-hoc test results are listed in Table S6.

MLCK is also a downsteam effector of RhoA whose activity is also suppressed in obscurin knockdown cells [11]. Remarkably, inhibition of MLCK via the pharmacological drug ML7 drastically reduced the ability of obscurin knockdown cells to randomly migrate away from their initial position, as evidenced by cell trajectories (Figure 6A). Indeed, migration speeds of ML7-treated obscurin knockdown cells significantly decreased across all substrate stiffnesses ranging from 4 to 280 kPa (Figure 6B; Table S6). Moreover, inhibition of MLCK in scramble control cells significantly reduced migration speed on 13 kPa and 280 kPa, but had no effect on cell speed on 4 kPa (Figure 6B; Table S6).

**Figure 7:**
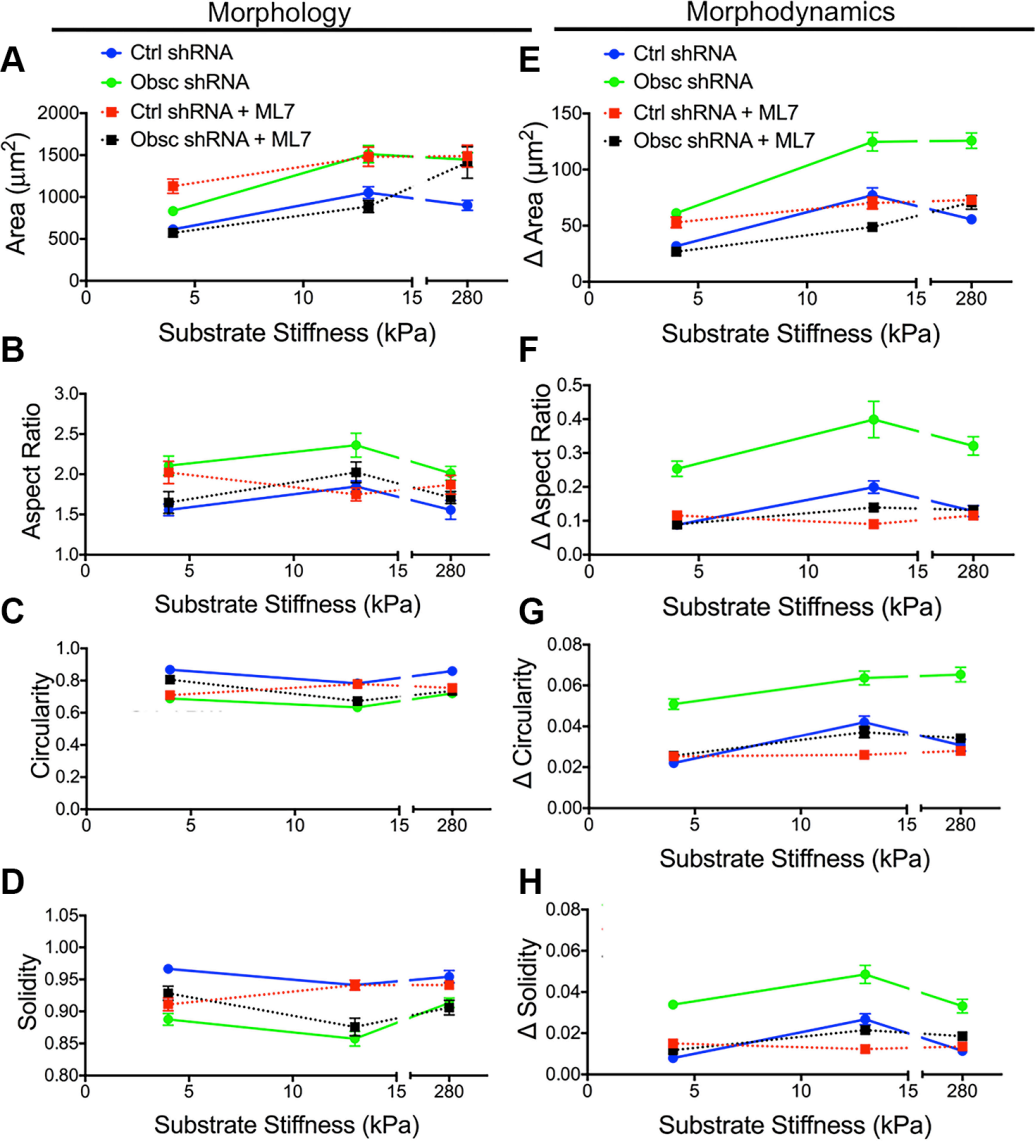
Morphology and morphodynamics of MCF10A Ctrl shRNA and Obsc shRNA following MLCK inhibition with ML7 Comparison of the various morphology parameters, including spreading area (**A**), aspect ratio (**B**), circularity (**C**) and solidity (**D**), between untreated and ML7-treated MCF10A Ctrl shRNA and Obsc shRNA cells across different substrate stiffness. Comparison of the change in morphology parameters (morphodynamics), including spreading area (**E**), aspect ratio (**F**), circularity (**G**) and solidity (**H**), between untreated and ML7-treated MCF10A Ctrl shRNA and Obsc shRNA across different substrate stiffness. Full ANOVA and Games-Howell post-hoc test results are listed in Tables S7 and S8.

Phase contrast images were used to evaluate the morphology of scramble control and obscurin knockdown cells following ML7 treatment (Figure 6C). Pharmacological treatment with ML7 significantly increased cell area in scramble control cells on 4 kPa and 280 kPa, similar to the effects of obscurin knockdown (Figure 7A; Table S7). Meanwhile, there was minimal impact of ML7 treatment on cell aspect ratio, circularity, or solidity on 13 kPa and 280 kPa; however, on softer 4 kPa substrates, aspect ratio significantly increased while circularity and solidity decreased, also similar to the effects of obscurin knockdown on that substrate (Figure 7B–7D; Table S7). Notably, treatment with ML7 significantly reduced the changes in all morphological parameters in obscurin knockdown cells (Figure 7E–7H; Table S8). It is possible that this decrease in morphodynamics may account for the significant decrease in cell migration speed and loss of dependence of cell migration on substrate stiffness caused by ML7 treatment in obscurin knockdown cells (Figure 6B; Table S6). We therefore postulate that inhibition of MLCK via ML7 acts differently on scramble control cells, in comparison with loss of MLCK activity via obscurin knockdown. However, in both cases, increased morphodynamics correlates with increased migratory potential and vice versa, at least in the range of stiffnesses tested.

### Loss of giant obscurins reduces focal adhesion area and traction stresses

**Figure 8:**
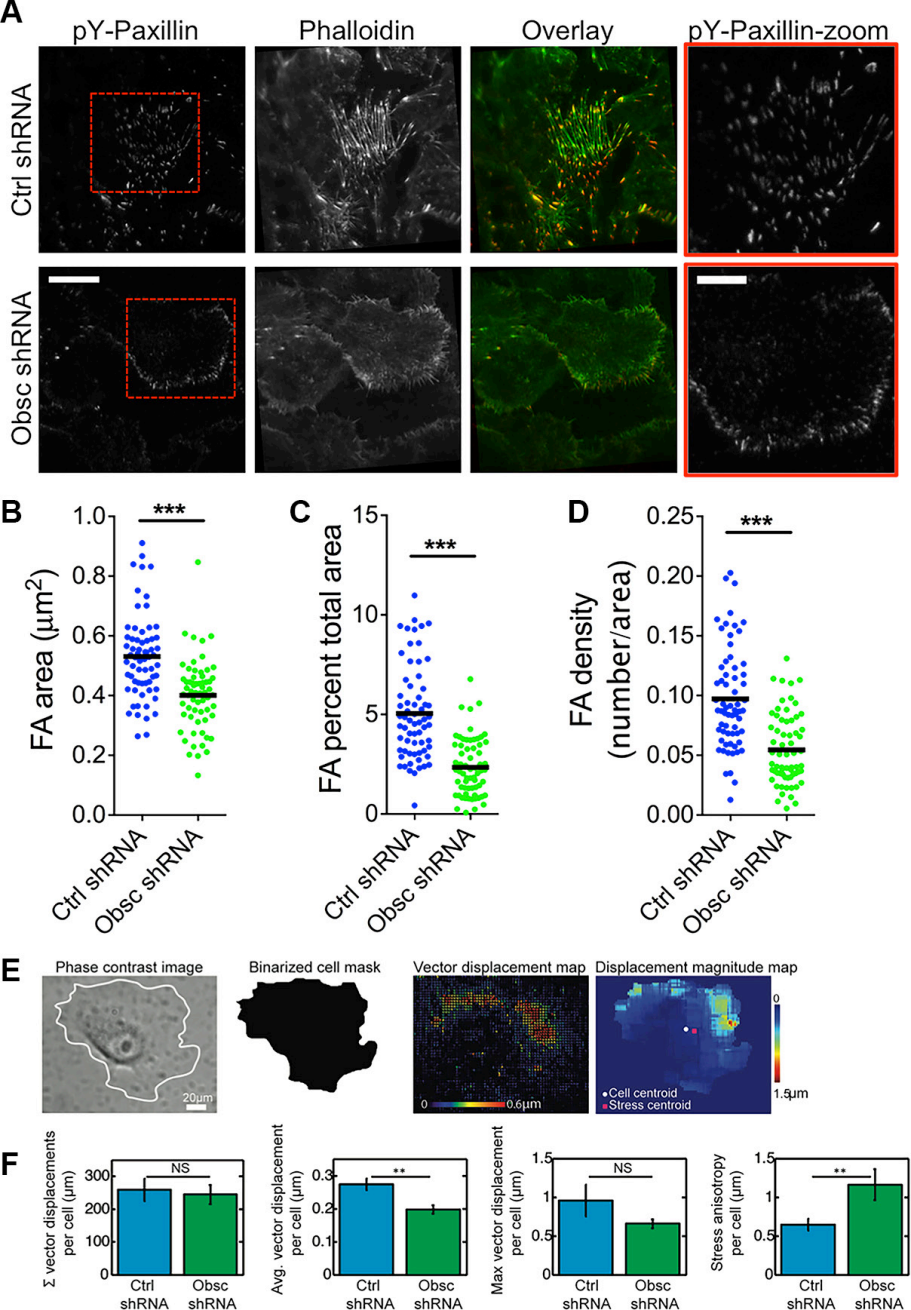
Loss of giant obscurins decreases focal adhesion area and density, and reduces traction forces in MCF10A breast epithelial cells (**A**) Representative total internal reflection fluorescence (TIRF) microscopy images of MCF10A Ctrl shRNA and Obsc shRNA cells plated on collagen-coated glass surface and stained with Alexa Fluor 488-conjugated phalloidin and immunostained with pY-paxillin to visualize actin and focal adhesions, respectively. Overlay images show actin in green and focal adhesions in red. Red boxes illustrate a zoomed in section of the pY-paxillin stain. (Scale bar represents 20 μm for all full-size images and 10 μm for the zoomed images) Comparison of the focal adhesion area (**B**), percentage of total cell area occupied for focal adhesions (**C**) and focal adhesion density (**D**) between MCF10A Ctrl shRNA and Obsc shRNA (***p < 0.001 using a paired Student's *t*-test). (**E**) Schematics of the analysis of the traction force microscopy experiment. Phase contrast image of the cell grown on 8 kPa polyacrylamide gels embedded with fluorescence beads was used to create a mask to delineate the boundaries of the cell. This mask was then applied to the vector map of bead displacements and the various traction stress parameters were calculated per single cell. (**F**) Comparison of the total summation vector displacements per cell, average vector displacement per cell, maximum vector displacement per cell and stress anisotropy per cell between MCF10A Ctrl shRNA and Obsc shRNA (**p < 0.01 using a paired Student's *t*-test.).

We hypothesize that the decrease of RhoA activity as a result of obscurin knockdown is accompanied by decreased myosin II activity. Myosin II activity promotes tyrosine-phosphorylation of paxillin [33], and thus paxillin-positive focal adhesions have been used as an indicator of myosin II activity [34]. Therefore, we used total internal reflection fluorescence (TIRF) microscopy to image pY-paxillin-stained focal adhesions in both scramble control and obscurin knockdown cells (Figure 8A). As we predicted, obscurin knockdown resulted in a decrease in focal adhesion area (Figure 8B), percentage of the total cell area occupied by focal adhesions (Figure 8C), and focal adhesion density (Figure 8D).

Decreased RhoA activity as well as decreased pY-paxillin staining would likely be associated with lower overall levels of cell contractility (i.e., in obscurin knockdown cells). To directly measure cellular contractility, we used traction force microscopy and quantified the cell traction-induced displacements of fluorescent beads within an 8 kPa polyacrylamide gel. Vector maps were used to visualize displacements of beads as a result of the cell traction forces (Figure 8E). Notably, the average vector displacement was significantly lower for obscurin knockdown cells, indicating overall lower traction forces in these cells (Figure 8F). Stress anisotropy, defined as the distance between the cell's geometric centroid and the stress centroid, was significantly higher for obscurin knockdown cells (Figure 8F), indicating that forces are more polarized for these cells in comparison with scramble controls.

## DISCUSSION

In this study, we demonstrated that knockdown of giant obscurins from MCF10A breast epithelial cells via obscurin shRNA increases cell morphodynamics and migration, while decreasing traction forces and focal adhesion size and density. In order to evaluate whether obscurins play a role in mechanosensing, we plated MCF10A scramble control or obscurin knockdown cells onto fibronectin-coated polyacrylamide gels and found that obscurin knockdown increases MCF10A mechanosensitivity in the 0.4-4 kPa range. These results build upon the growing evidence that loss of giant obscurins from breast epithelial cell lines or primary breast tissue samples, is involved in multiple steps of the metastatic cascade: from epithelial-to-mesenchymal transition, to migration and invasion, to the formation of secondary tumors, and, as we demonstrate here, to sensing heterogeneous mechanical environments.

Cells are influenced by input from mechanical cues, which can be generated by the cells themselves (endogenous) or by the matrix in which they reside (exogenous). This matrix can be either the native tissue or a tissue-engineered construct. During the metastatic cascade, tumor cells must navigate through complex microenvironments of different mechanical properties [1]. Thus, biomaterial-based approaches for cancer therapeutics as well as *in vitro* culture systems for tumor cells must account for this heterogeneity in mechanical input. The stiffness of the cellular microenvironment varies throughout different tissues *in vivo* [2], and matrix stiffness is a key parameter in biomaterial design, since this mechanical cue can direct cell motility [3, 7, 15, 35–37], cell-substrate adhesion [7], cell-cell adhesion [35, 38], cell mechanical properties [39–41], proliferation [42], differentiation [43], cytoskeletal organization [4, 7, 31], traction forces [6, 38, 44], and biochemical signaling [7].

Recent work from our lab has implicated giant obscurins in breast cancer progression and metastasis [10–12]. Notably, loss of giant obscurins from normal breast epithelial cells leads to epithelial-to-mesenchymal transition, increased invasiveness, increased migration, altered actin cytoskeletal structures, increased actin cytoskeletal dynamics, and formation of primary and metastatic lesions *in vivo* [12]. Our recent work has also shown that depletion of giant obscurins from MCF10A cells (both attached and suspended) significantly reduces RhoA activity and thus phosphorylation of RhoA downstream effectors, including myosin light chain phosphatase, myosin light chain (MLC), lim kinase, and cofilin [11]. Rho GTPases, including RhoA, regulate stress fibers and focal adhesions [29], two structures whose assembly is tightly controlled by matrix stiffness. Herein, we demonstrate that loss of giant obscurins from MCF10A breast epithelial cells alters cell mechanosensitivity during migration, and our experimental results suggest that this occurs via the RhoA pathway.

Scramble control and obscurin knockdown cells on the softest gels with Young's modulus 0.4 kPa were mostly unattached, with low spreading areas and circularity and solidity close to 1. Healthy breast epithelial tissue has an elastic modulus of ˜167 Pa [27], which is softer than the softest polyacrylamide gel we tested. Most likely, the extracellular matrix surrounding breast epithelial cells contains additional cues (e.g., growth factors, extracellular matrix proteins, biochemical cues, etc.) that promote their adherence in this soft mechanical environment *in vivo*. Breast tumors are significantly stiffer than healthy breast tissue, with an elastic modulus of ˜4 kPa [27]. In our experiments, on gels of stiffness 4 kPa and above, we observed significant differences in cell morphology parameters between scramble control and obscurin knockdown cells, with a notable difference being the increase in solidity (indicating increased protrusiveness) in obscurin knockdown cells. Indeed, *in vivo*, protrusions known as pseudopods are important for tumor cell invasion through the basement membrane, extracellular matrix, epithelial, and endothelial layers at various stages during the metastatic cascade [45].

We and others have shown that RhoA or ROCK1 inhibition or depletion can lead to cell elongation and defective tail retraction [32, 46]. Herein, we show that inhibition of ROCK via Y27632 leads to elongated scramble control cells with larger aspect ratios, and also renders both scramble control and obscurin knockdown cells unable to completely retract their tails. Obscurin knockdown cells (where RhoA activation is markedly diminished [11]) also inherently display more elongated morphologies than scramble control cells, though inhibition of the ROCK pathway via Y27632 does not further increase elongation in obscurin knockdown cells.

While morphology alone has been linked to cell motility and other functions, we hypothesized that morphodynamics, i.e., shape changes over time, were also important in governing cell migration. Indeed, we found that morphodynamics were significantly increased in obscurin knockdown cells, in comparison with scramble control cells, on gels 1 kPa and stiffer. Notably, this is the same stiffness where we also began to observe statistical differences between scramble control and obscurin knockdown cells in cell migration parameters (i.e., speed and diffusion coefficient). On all substrate stiffnesses tested, inhibition of ROCK via Y27632 significantly increased morphodynamics of scramble control cells, which correlated with increased migration speeds. Together, these results suggest that the reduction in RhoA activation that occurs in obscurin knockdown cells increases MCF10A morphodynamics, which enables dynamic cell migration patterns. Further inhibition of ROCK in obscurin-knockdown cells fails to affect cell migration speed on 4, 13 or 280 kPa, suggesting there exists an optimal cell contractility level necessary for maximal migration, which can be achieved either through optimal internal ROCK activity or through external mechanical cues such as matrix stiffness. We postulate that scramble control MCF10A cells, with a higher ROCK-mediated contractility level, are on the down slope of the biphasic curve that relates cell migration and cell contractility (Figure 4C), while obscurin knockdown cells are on the up slope. Treating scramble control cells with Y27632 reduces ROCK-mediated cell contractility and shifts their migration to the up slope of the biphasic curve, thereby increasing their migration speed. Meanwhile, treating obscurin knockdown cells (originally with lower ROCK-mediated contractility) with Y27632 may have little effect on contractility and thus little or no effect on cell migration. In addition, multiple cell lines have been shown to exhibit biphasic relationships between cell migration speed and substrate stiffness [3, 37, 47], which corroborates the idea that there may exist an optimal cell contractility level necessary for maximal migration. Indeed, we observed a peak in migration (both speed and diffusion coefficient) in scramble control cells on 13 kPa, indicative of a biphasic behavior with substrate stiffness.

MLCK is also a downstream effector of RhoA whose activity is also suppressed in obscurin knockdown cells [11]. MLCK seems also to be involved in the correlation between cell morphodynamics and cell migration, since inhibition of MLCK via ML7 significantly reduced morphodynamics of scramble control cells on 13 kPa and 280 kPa, and of obscurin knockdown cells on all stiffnesses tested, which correlated with a drastic reduction in cell migration speed and exploration area. The divergent effects of ROCK inhibition (leading to increased migration) and MLCK inhibition (leading to decreased migration) in MCF10A cells with control shRNA are consistent with previous reports demonstrating distinct roles of ROCK and MLCK in the regulation of membrane protrusions and focal adhesion dynamics during fibroblast migration [48]. These results seem to be linked to the spatial regulation of MLC phosphorylation. In fibroblasts, ROCK inhibition blocks MLC phosphorylation in the center of the cell, but not at the periphery, while MLCK inhibition blocks MLC phosphorylation at the cell periphery, but not in the center. As a result, ROCK-inhibited fibroblasts display mature focal adhesions at the periphery (but not in the center) and migrate faster and straighter than control cells, while MLCK-inhibited fibroblasts display mature focal adhesions in the center (but not at the periphery) and migrate less effectively [48]. This distinct role for ROCK and MLCK in fibroblast migration is consistent with our results for MCF10A with control shRNA and may provide insight into the different effects these molecules play during morphodynamics and migration of MCF10A cells.

Focal adhesions are the main anchoring points between the cell and its ECM. These structures link transmembrane integrins, which bind proteins within the ECM, to the actin cytoskeleton, and have been shown to play a role in many aspects of cell mechanobiology. In general, focal adhesions may be stable with low turnover rates, promoting cell attachment, spreading, and slow migration; or, in contrast, focal adhesions may be dynamic, with high turnover rates, facilitating cell migration [49, 50]. Integrin binding in cells can trigger RhoA-mediated signaling pathways, and thus we hypothesized that obscurin knockdown cells, where RhoA activity is diminished, would demonstrate fewer (and smaller) focal adhesion structures in comparison with scramble control cells. Indeed, total internal reflection fluorescence imaging of tyrosine-phosphorylated paxillin (pY-paxillin)-stained MCF10A cells corroborated this hypothesis. Obscurin knockdown MCF10A cells had less dense and smaller focal adhesions in comparison with scramble control cells, which suggests increased turnover of focal adhesions, and concomitant enhancement of morphodynamics and cell migration. We have previously shown that obscurin knockdown increases the mobile fraction of actin at cell-cell contacts, and also in migrating cells [12]; since focal adhesions connect to and stabilize the actin cytoskeleton, it makes sense that downregulation of focal adhesion structures, as we observe here, correlates with increased actin dynamics (i.e., in obscurin knockdown cells).

Tyrosine phosphorylation of paxillin in focal adhesions can also be used to evaluate cell contractile activity [34], since pY-paxillin depends on myosin II activity [33]. Therefore, the decrease in pY-paxillin-positive focal adhesion size and density in obscurin knockdown cells would suggest lower traction stresses in these cells. In agreement with our focal adhesion analysis, we found that traction stresses were significantly decreased in obscurin knockdown cells. It has been suggested that weaker adhesions allow cells to move along their substrate without exerting significant force on the substrate to overcome resisting adhesive forces [51]. However, some adhesion and traction forces are obviously necessary, since lack of adherence, as on our 0.4 kPa gel, prevents spreading and migration.

Future work could address some of the limitations of the current study. First, this work only considers the effects of giant obscurins on the mechanobiological response of MCF10A breast epithelial cells to matrix stiffness. These cells were chosen due to the mounting evidence our labs have found regarding the role of giant obscurins in breast tumor formation and metastasis [10–12, 26, 52]. The *OBSCN* gene was identified as one of 189 “candidate cancer genes” in both breast and colorectal cancers due to its high mutational frequency [24], and therefore future studies could examine whether obscurins play a similar role in promoting metastasis and mechanosensing in the context of colorectal cancer. An additional limitation of the study stems from the use of pharmacological inhibitors (i.e., Y27632 and ML7) rather than genetically modifying ROCK or MLCK activity. Knocking down or knocking out ROCK or MLCK would be of particular interest due to the potential off target effects of pharmacological inhibitors. For example, the secondary targets of Y27632 include most members of the AGC subfamily [53]. However, because we only observed statistically significant differences in cell migration in scramble control cells, and not obscurin knockdown cells following Y27632 treatment, it is likely that effects on secondary targets may have been minimal compared to primary effects of ROCK 1 inhibition.

In summary, we have demonstrated that loss of giant obscurins alters cell morphology, increases morphodynamics and mechanosensitivity, and affects focal adhesion morphology and traction forces. Together, our results indicate that loss of giant obscurins facilitates cell migration through heterogeneous microenvironments of varying stiffness by altering cell mechanobiology via RhoA-mediated effects. Loss of giant obscurins during the initial steps of the metastatic cascade could enable enhanced mechanosensing of the heterogeneous tumor environment, allowing the cells to migrate out from the stiffened tumor matrix and into the softer surrounding healthy tissue, into a nearby vascular structure, and later, into secondary locations after extravasation. Based on our results, we speculate that the cell's ability to sense and migrate within these mechanically heterogeneous environments relies on efficient morphodynamics and formation of protrusive structures, which are enhanced upon loss of giant obscurins and concomitant deactivation of the RhoA/ROCK pathway.

## MATERIALS AND METHODS

### Preparation of polyacrylamide substrates

Thin polyacrylamide gels were attached to glass coverslips (22 × 22 mm, Fisher Scientific) or to 35 mm glass-bottom dishes via the method originally described by Wang and Pelham [54] and described in our previous publications [3, 4]. Gel compositions used herein included 15% acrylamide + 1.2% bis (280 kPa), 8% acrylamide + 0.2% bis (13 kPa), 5% acrylamide + 0.3% bis (8 kPa), 8% acrylamide + 0.1% bis (7 kPa), 8% acrylamide + 0.04% bis (4 kPa), 3% acrylamide + 0.2% bis (1 kPa), and 3% acrylamide + 0.05% bis (0.42 kPa). Gels were approximately 80 μm thick after polymerization and were subsequently coated with 50 μg/ml fibronectin (Sigma-Aldrich) or collagen type I (for traction force microscopy) as previously described [3]. Dynamic mechanical analysis and atomic force microscopy were used to measure Young's moduli of the gels, and immunofluorescence was used to characterize surface-bound fibronectin [3, 55]. For experiments on glass, PDMS with bored holes was bound to a glass coverslip via plasma treatment, and the glass was coated with 50 μg/ml fibronectin or collagen type I (BD Biosciences) for 1 h at 37°C. Gels were thoroughly washed with PBS and soaked with media prior to cell plating.

### Cell culture

MCF10A stable clones expressing either obscurin shRNA or control shRNA plasmids were generated and cultured as previously described [10]. Cells (1 × 10^5^ total) were plated onto the ECM-coated polyacrylamide gels and grown overnight. For experiments involving drug treatments, cells were incubated with media containing 25 μM ML7 (Sigma-Aldrich) or 30 μM Y27632 (Sigma-Aldrich) for 1 h at 37°C prior to microscopy. Drugs were maintained in the cell culture media during microscopy.

### Generation of protein lysates, Western blotting, and Rho GTPase pulldown

For Western Blotting, MCF10A cells expressing control shRNA or obscurin shRNA (targeting Ig domain 24) were grown to ˜70% confluence, and fresh complete media was added for 24 hours before protein lysates were prepared on ice in *radioimmuno*-precipitation assay (RIPA) buffer supplemented with a cocktail of protease inhibitors (Roche) and phosphatase inhibitors (200 nM Imidazole, 100 mM Sodium Flouride, 115 mM Sodium Molybdate, 100 mM Sodium Orthovanadate, 400 mM Sodium Tartrate Dihydrate, 100 mM b-Glycerophosphate, 100 mM Sodium Pyrophosphate, and 10 mM EGTA), as previously described [56]. Equal amounts of protein lysates were electrophoresed on SDS-NuPAGE gel (Invitrogen), transferred to nitrocellulose membranes, and probed with a rabbit polyclonal obscurin-COOH antibody (600 ng/ml) [57]. β-actin was used as a loading control. Alkaline phosphatase-conjugated anti-rabbit IgG (1:3000; Jackson ImmunoResearch) was used, and a chemiluminescence detection kit (Applied Biosystems) was used to visualize immunoreactive bands. Control and obscurin shRNA MCF10A cells were grown as for Western blots above, before they were collected, lysed, and used for Rho GTPase pulldowns as per the manufacturer's protocols (Cytoskeleton, Inc.). HSP90 served as a loading control.

### Live cell microscopy and analysis

Six-well plates containing coverslips with cells on gels were mounted onto the stage of a Nikon T2i inverted microscope. Cells were maintained at 37°C with 5% CO_2_ and humidity within a stage top incubator (Tokai Hit Co., Japan). At least 5 locations per sample were selected for imaging. Phase contrast images were recorded every 10 min for 10 h at all positions using stage automation and Nikon Elements software. Experiments were repeated ≥ 3 times and image time series were imported to ImageJ (National Institutes of Health, Bethesda, MD). The first 5 h of the timelapse was chosen for analysis. Outlines of cells were drawn for every time point and morphology parameters (e.g., area, aspect ratio, circularity, solidity) were measured using ImageJ. Cell aspect ratio was defined as the ratio of the major axis length to the minor axis length. Cell circularity was defined as $\frac{4\text{πA}}{p^{2}}$, where *A* is the area and *P* is the perimeter of the cell projection in phase contrast images. Cell solidity was defined as $\frac{A_{cell}}{A_{convex}}$, where *A*_cell_ is the cell area and *A*_convex_ is the convex area of the cell. Changes in these morphology parameters were quantified by calculating the difference between the morphology parameter at two consecutive time points. For a given cell, the changes in morphology parameters between all 10-min time steps were calculated and averaged. Instantaneous cell velocity and diffusion coefficients were calculated from cell trajectories using a custom-written Matlab code, as previously described [3].

### Total internal reflection fluorescence microscopy

Total internal reflection fluorescence (TIRF) microscopy was used to characterize focal adhesions as previously described [34]. MCF10A cells were plated onto collagen I-coated glass coverslips, fixed in 3.7% formaldehyde for 10 min, permeabilized in 0.5% Triton X-100 for 5 min, and blocked in 2.5% BSA for 1 h. Cells were incubated in an antibody against pY-paxillin (1:100; Cell Signaling Technology) for 2 h, followed by an Alexa Fluor 568 secondary antibody (1:100; Invitrogen) and Alexa Fluor 488-conjugated phalloidin (1:500; Molecular Probes) for 1 h. Cells were washed with PBS in between each step. TIRF microscopy was completed using an inverted 3i Marianas microscope (Intelligent Imaging Innovations, Inc.) equipped with dual electron multiplying charge-coupled device cameras (Cascade II: 512; Photometrics) for simultaneous two-channel TIRF acquisition, along with a 100×/NA 1.45 oil immersion objective and Slidebook 8.0 software (Intelligent Imaging Innovations, Inc.). TIRF images were processed in ImageJ and focal adhesion parameters (individual focal adhesion area, fraction of total cell area, and density) were quantified as previously described [34, 58, 59]. Total cell area was determined using the Alexa Fluor 488-conjugated phalloidin images. Experiments were performed in triplicates, and at least 20 cells were analyzed for each experiment, for a total of ≥ 60 cells per condition.

### Traction force microscopy

For traction force microscopy experiments, 8 kPa polyacrylamide gels were prepared (as described above) with 0.2 μm carboxylate-modified FluoSpheres (Life Technologies) embedded within the substrate. Subsequently, substrates were allowed to equilibrate in warm cell culture media at 37°C for 10 min. Once equilibrated, cells were seeded at low density onto the substrates and allowed to spread for 24 h. Using a Nikon T300 epifluorescent microscope equipped with a motorized stage, 20× plan fluor lens, and a Nikon digital sight DS-Qi1MC CCD camera, phase contrast images of the cells and the corresponding fluorescence images of the apical surface of the bead array per position were acquired and labeled as the ‘stressed state’. After image acquisition, media was aspirated without moving the dish, and cells were washed once with PBS and then incubated in 0.25% trypsin-EDTA (Gibco) for 10 min. After ensuring that all cells had detached from the substrate surface, the substrate was washed with PBS, and fluorescence images of the bead array, corresponding to the previously defined positions, were acquired and labeled as the ‘unstressed state’.

Stressed and unstressed images were analyzed using a custom Matlab program to quantify the spatially defined traction stresses exerted per single cell. Briefly, stressed and unstressed images were imported and signal intensity was amplified via a low-pass filter. Using cross-correlation analysis and image shift corrections, the two images were mapped onto each other and the local stresses exerted per cell were quantified as the displacement of the beads beneath the cells of interest. The bead displacement lengths were rendered as a vector map and the phase contrast image of the cell was used to create a mask to delineate the boundaries of the cell. This mask was applied to the vector map of bead displacements and the various traction stress parameters were calculated per single cell. The summation vector displacement per cell was defined as the sum of the vector lengths within the cell region of interest, which provides insight into the total stress exerted per single cell. The stress anisotropy was defined as the vector difference between the geometric cell centroid and the stress centroid (spatial vector-weighted center per cell), which provides insight into the stress polarity/imbalance per cell.

### Statistical analysis

At least 3 independent trials were conducted for each experiment, for a total of at least 50 cells per condition. Statistical significance (i.e., *P* < 0.05) was determined between pairs of data using a paired Student's *t*-test, or among groups of data using one-way ANOVA. The Games-Howell post-hoc test was used at 95% confidence for multiple comparisons following ANOVA, since most groups contained unequal variance. Results from ANOVA and post-hoc tests are included in Tables S1–S8. All measurements reported here are in the format mean ± S.E.M. Graphpad Prism (Graphpad) was used to generate plots and Minitab was used for statistical analyses.

## SUPPLEMENTARY MATERIALS TABLES


