# Supplementary material for: Loss of giant obscurins alters breast epithelial cell mechanosensing of matrix stiffness
Source: Oncotarget. 2016 Aug 1;8(33):54004–20. doi: 10.18632/oncotarget.10997 (PMC5589558; doi:10.18632/oncotarget.10997)
Supplement: Supplementary file 1 [file oncotarget-08-54004-s001.pdf]

Loss of giant obscurins alters breast epithelial cell mechanosensing of matrix stiffness

Supplementary Materials

Supplementary Table S1: Following ANOVA, Games-Howell post-hoc tests were completed for pairwise comparisons between groups of data

Δ Area (Figure 2D)

**Games-Howell Pairwise Comparisons**<br>*Grouping Information Using 95% Confidence*<br>*Means that do not share a letter are significantly different.*	
0.4 kPa - Ctrl shRNA	F
0.4 kPa - Obsc shRNA	F
1 kPa - Ctrl shRNA	E F
1 kPa - Obsc shRNA	C D E
4 kPa - Ctrl shRNA	F
4 kPa - Obsc shRNA	C D
7 kPa - Ctrl shRNA	D E
7 kPa - Obsc shRNA	B
13 kPa - Ctrl shRNA	B C
13 kPa - Obsc shRNA	A
280 kPa - Ctrl shRNA	C D E
280 kPa - Obsc shRNA	A

Δ Aspect ratio (Figure 2E)

**Games-Howell Pairwise Comparisons**<br>*Grouping Information Using 95% Confidence*<br>*Means that do not share a letter are significantly different.*	
0.4 kPa - Ctrl shRNA	D E
0.4 kPa - Obsc shRNA	E
1 kPa - Ctrl shRNA	D E
1 kPa - Obsc shRNA	B C
4 kPa - Ctrl shRNA	D E
4 kPa - Obsc shRNA	A B
7 kPa - Ctrl shRNA	D
7 kPa - Obsc shRNA	A
13 kPa - Ctrl shRNA	B C
13 kPa - Obsc shRNA	A
280 kPa - Ctrl shRNA	C D
280 kPa - Obsc shRNA	A

Δ Circularity (Figure 2F)

**Games-Howell Pairwise Comparisons**<br>*Grouping Information Using 95% Confidence*<br>*Means that do not share a letter are significantly different.*	
0.4 kPa - Ctrl shRNA	G
0.4 kPa - Obsc shRNA	F G
1 kPa - Ctrl shRNA	E F
1 kPa - Obsc shRNA	B C D
4 kPa - Ctrl shRNA	E F
4 kPa - Obsc shRNA	A B
7 kPa - Ctrl shRNA	D E
7 kPa - Obsc shRNA	A
13 kPa - Ctrl shRNA	B C
13 kPa - Obsc shRNA	A
280 kPa - Ctrl shRNA	C D E
280 kPa - Obsc shRNA	A

Δ Solidity (Figure 2G)

**Games-Howell Pairwise Comparisons**<br>*Grouping Information Using 95% Confidence*<br>*Means that do not share a letter are significantly different.*	
0.4 kPa - Ctrl shRNA	F
0.4 kPa - Obsc shRNA	E F
1 kPa - Ctrl shRNA	C D
1 kPa - Obsc shRNA	C
4 kPa - Ctrl shRNA	D E F
4 kPa - Obsc shRNA	B
7 kPa - Ctrl shRNA	D
7 kPa - Obsc shRNA	A B
13 kPa - Ctrl shRNA	C
13 kPa - Obsc shRNA	A
280 kPa - Ctrl shRNA	C D E
280 kPa - Obsc shRNA	A B

Grouping information is shown in all tables using a 95% confidence level. Data correspond to main text figure indicated at the top of each subtable. *Groups that do not share a letter are significantly different from each other.*

Supplementary Table S2: Following ANOVA, Games-Howell post-hoc tests were completed for pairwise comparisons between groups of data

Speed (Figure 3C)

**Games-Howell Pairwise Comparisons** *Grouping Information Using 95% Confidence Means that do not share a letter are significantly different.*	
0.4 kPa - Ctrl shRNA	D E
0.4 kPa - Obsc shRNA	D E
1 kPa - Ctrl shRNA	D E
1 kPa - Obsc shRNA	B C
4 kPa - Ctrl shRNA	E
4 kPa - Obsc shRNA	B
7 kPa - Ctrl shRNA	C D
7 kPa - Obsc shRNA	A
13 kPa - Ctrl shRNA	B
13 kPa - Obsc shRNA	A
280 kPa - Ctrl shRNA	B C D
280 kPa - Obsc shRNA	A

Diffusion coefficient (Figure 3D)

**Games-Howell Pairwise Comparisons** *Grouping Information Using 95% Confidence Means that do not share a letter are significantly different.*	
0.4 kPa - Ctrl shRNA	E
0.4 kPa - Obsc shRNA	E
1 kPa - Ctrl shRNA	E
1 kPa - Obsc shRNA	C D
4 kPa - Ctrl shRNA	E
4 kPa - Obsc shRNA	B C
7 kPa - Ctrl shRNA	D E
7 kPa - Obsc shRNA	A B
13 kPa - Ctrl shRNA	C D
13 kPa - Obsc shRNA	A
280 kPa - Ctrl shRNA	D E
280 kPa - Obsc shRNA	A

Grouping information is shown in all tables using a 95% confidence level. Data correspond to main text figure indicated at the top of each subtable. *Groups that do not share a letter are significantly different from each other.*

Supplementary Table S3: Following ANOVA, Games-Howell post-hoc tests were completed for pairwise comparisons between groups of data

Speed (Figure 4B)

Games-Howell Pairwise Comparisons<br>*Grouping Information Using 95% Confidence*<br>*Means that do not share a letter are significantly different.*	
4 kPa - Ctrl shRNA	E
4 kPa - Obsc shRNA	C D
13 kPa - Ctrl shRNA	B C D
13 kPa - Obsc shRNA	A
280 kPa - Ctrl shRNA	D
280 kPa - Obsc shRNA	A
4 kPa - Ctrl shRNA (with Y27632)	D
4 kPa - Obsc shRNA (with Y27632)	D
13 kPa - Ctrl shRNA (with Y27632)	A
13 kPa - Obsc shRNA (with Y27632)	A B C
280 kPa - Ctrl shRNA (with Y27632)	A
280 kPa - Obsc shRNA (with Y27632)	A B C

Grouping information is shown in all tables using a 95% confidence level. Data correspond to main text figure indicated at the top of each subtable. *Groups that do not share a letter are significantly different from each other.*

Supplementary Table S4: Following ANOVA, Games-Howell post-hoc tests were completed for pairwise comparisons between groups of data

Area (Figure 5A)

Games-Howell Pairwise Comparisons<br>*Grouping Information Using 95% Confidence*<br>*Means that do not share a letter are significantly different.*	
4 kPa - Ctrl shRNA	E
4 kPa - Obsc shRNA	D
13 kPa - Ctrl shRNA	C D
13 kPa - Obsc shRNA	A B
280 kPa - Ctrl shRNA	D
280 kPa - Obsc shRNA	B
4 kPa - Ctrl shRNA (with Y27632)	D
4 kPa - Obsc shRNA (with Y27632)	A B C
13 kPa - Ctrl shRNA (with Y27632)	A B
13 kPa - Obsc shRNA (with Y27632)	A B
280 kPa - Ctrl shRNA (with Y27632)	A
280 kPa - Obsc shRNA (with Y27632)	A

Aspect ratio (Figure 5B)

Games-Howell Pairwise Comparisons<br>*Grouping Information Using 95% Confidence*<br>*Means that do not share a letter are significantly different.*	
4 kPa - Ctrl shRNA	D
4 kPa - Obsc shRNA	A B C
13 kPa - Ctrl shRNA	B C D
13 kPa - Obsc shRNA	A B
280 kPa - Ctrl shRNA	C D
280 kPa - Obsc shRNA	A B C
4 kPa - Ctrl shRNA (with Y27632)	A
4 kPa - Obsc shRNA (with Y27632)	A B
13 kPa - Ctrl shRNA (with Y27632)	A B C
13 kPa - Obsc shRNA (with Y27632)	A B C
280 kPa - Ctrl shRNA (with Y27632)	A B C
280 kPa - Obsc shRNA (with Y27632)	B C D

Circularity (Figure 5C)

Games-Howell Pairwise Comparisons<br>*Grouping Information Using 95% Confidence*<br>*Means that do not share a letter are significantly different.*	
4 kPa - Ctrl shRNA	A
4 kPa - Obsc shRNA	D E
13 kPa - Ctrl shRNA	B C
13 kPa - Obsc shRNA	D E F
280 kPa - Ctrl shRNA	A B
280 kPa - Obsc shRNA	C D
4 kPa - Ctrl shRNA (with Y27632)	H
4 kPa - Obsc shRNA (with Y27632)	G H
13 kPa - Ctrl shRNA (with Y27632)	G H
13 kPa - Obsc shRNA (with Y27632)	F G H
280 kPa - Ctrl shRNA (with Y27632)	E F G
280 kPa - Obsc shRNA (with Y27632)	D E

Solidity (Figure 5D)

Games-Howell Pairwise Comparisons<br>*Grouping Information Using 95% Confidence*<br>*Means that do not share a letter are significantly different.*	
4 kPa - Ctrl shRNA	A
4 kPa - Obsc shRNA	C D
13 kPa - Ctrl shRNA	B
13 kPa - Obsc shRNA	D E
280 kPa - Ctrl shRNA	A B
280 kPa - Obsc shRNA	B C
4 kPa - Ctrl shRNA (with Y27632)	G
4 kPa - Obsc shRNA (with Y27632)	F G
13 kPa - Ctrl shRNA (with Y27632)	F G
13 kPa - Obsc shRNA (with Y27632)	F G
280 kPa - Ctrl shRNA (with Y27632)	E F
280 kPa - Obsc shRNA (with Y27632)	C D E

Grouping information is shown in all tables using a 95% confidence level. Data correspond to main text figure indicated at the top of each subtable. *Groups that do not share a letter are significantly different from each other.*

Supplementary Table S5: Following ANOVA, Games-Howell post-hoc tests were completed for pairwise comparisons between groups of data

Δ Area (Figure 5E)

**Games-Howell Pairwise Comparisons**<br>*Grouping Information Using 95% Confidence*<br>*Means that do not share a letter are significantly different.*	
4 kPa - Ctrl shRNA	D
4 kPa - Obsc shRNA	C
13 kPa - Ctrl shRNA	B C
13 kPa - Obsc shRNA	A
280 kPa - Ctrl shRNA	C
280 kPa - Obsc shRNA	A
4 kPa - Ctrl shRNA (with Y27632)	C
4 kPa - Obsc shRNA (with Y27632)	B C
13 kPa - Ctrl shRNA (with Y27632)	A B
13 kPa - Obsc shRNA (with Y27632)	B
280 kPa - Ctrl shRNA (with Y27632)	A
280 kPa - Obsc shRNA (with Y27632)	A

Δ Aspect ratio (Figure 5F)

**Games-Howell Pairwise Comparisons**<br>*Grouping Information Using 95% Confidence*<br>*Means that do not share a letter are significantly different.*	
4 kPa - Ctrl shRNA	D
4 kPa - Obsc shRNA	A B
13 kPa - Ctrl shRNA	B C
13 kPa - Obsc shRNA	A
280 kPa - Ctrl shRNA	C D
280 kPa - Obsc shRNA	A
4 kPa - Ctrl shRNA (with Y27632)	B C
4 kPa - Obsc shRNA (with Y27632)	B C
13 kPa - Ctrl shRNA (with Y27632)	A B
13 kPa - Obsc shRNA (with Y27632)	A B
280 kPa - Ctrl shRNA (with Y27632)	A B
280 kPa - Obsc shRNA (with Y27632)	B C

Δ Circularity (Figure 5G)

**Games-Howell Pairwise Comparisons**<br>*Grouping Information Using 95% Confidence*<br>*Means that do not share a letter are significantly different.*	
4 kPa - Ctrl shRNA	F
4 kPa - Obsc shRNA	A B
13 kPa - Ctrl shRNA	B C D
13 kPa - Obsc shRNA	A
280 kPa - Ctrl shRNA	D E F
280 kPa - Obsc shRNA	A
4 kPa - Ctrl shRNA (with Y27632)	E F
4 kPa - Obsc shRNA (with Y27632)	C D E
13 kPa - Ctrl shRNA (with Y27632)	A B C
13 kPa - Obsc shRNA (with Y27632)	A B C
280 kPa - Ctrl shRNA (with Y27632)	A B
280 kPa - Obsc shRNA (with Y27632)	A B

Δ Solidity (Figure 5H)

**Games-Howell Pairwise Comparisons**<br>*Grouping Information Using 95% Confidence*<br>*Means that do not share a letter are significantly different.*	
4 kPa - Ctrl shRNA	E
4 kPa - Obsc shRNA	B C
13 kPa - Ctrl shRNA	D
13 kPa - Obsc shRNA	A
280 kPa - Ctrl shRNA	D E
280 kPa - Obsc shRNA	A B C
4 kPa - Ctrl shRNA (with Y27632)	C
4 kPa - Obsc shRNA (with Y27632)	C
13 kPa - Ctrl shRNA (with Y27632)	A
13 kPa - Obsc shRNA (with Y27632)	A B
280 kPa - Ctrl shRNA (with Y27632)	A B C
280 kPa - Obsc shRNA (with Y27632)	A B C

Grouping information is shown in all tables using a 95% confidence level. Data correspond to main text figure indicated at the top of each subtable. *Groups that do not share a letter are significantly different from each other.*

Supplementary Table S6: Following ANOVA, Games-Howell post-hoc tests were completed for pairwise comparisons between groups of data

Speed (Figure 6B)

**Games-Howell Pairwise Comparisons**<br>*Grouping Information Using 95% Confidence*<br>*Means that do not share a letter are significantly different.*	
4 kPa - Ctrl shRNA	D
4 kPa - Obsc shRNA	B
13 kPa - Ctrl shRNA	B
13 kPa - Obsc shRNA	A
280 kPa - Ctrl shRNA	B C
280 kPa - Obsc shRNA	A
4 kPa - Ctrl shRNA (with ML7)	D
4 kPa - Obsc shRNA (with ML7)	D
13 kPa - Ctrl shRNA (with ML7)	C D
13 kPa - Obsc shRNA (with ML7)	C
280 kPa - Ctrl shRNA (with ML7)	C
280 kPa - Obsc shRNA (with ML7)	B C

Grouping information is shown in all tables using a 95% confidence level. Data correspond to main text figure indicated at the top of each subtable. *Groups that do not share a letter are significantly different from each other.*

Supplementary Table S7: Following ANOVA, Games-Howell post-hoc tests were completed for pairwise comparisons between groups of data

Area (Figure 7A)

**Games-Howell Pairwise Comparisons**<br>*Grouping Information Using 95% Confidence*<br>*Means that do not share a letter are significantly different.*	
4 kPa - Ctrl shRNA	D
4 kPa - Obsc shRNA	C
13 kPa - Ctrl shRNA	B C
13 kPa - Obsc shRNA	A
280 kPa - Ctrl shRNA	C
280 kPa - Obsc shRNA	A
4 kPa - Ctrl shRNA (with ML7)	A B C
4 kPa - Obsc shRNA (with ML7)	D
13 kPa - Ctrl shRNA (with ML7)	A B
13 kPa - Obsc shRNA (with ML7)	C
280 kPa - Ctrl shRNA (with ML7)	A B
280 kPa - Obsc shRNA (with ML7)	A B C

Aspect ratio (Figure 7B)

**Games-Howell Pairwise Comparisons**<br>*Grouping Information Using 95% Confidence*<br>*Means that do not share a letter are significantly different.*	
4 kPa - Ctrl shRNA	D
4 kPa - Obsc shRNA	B C
13 kPa - Ctrl shRNA	B
13 kPa - Obsc shRNA	A
280 kPa - Ctrl shRNA	B C
280 kPa - Obsc shRNA	A
4 kPa - Ctrl shRNA (with ML7)	B C
4 kPa - Obsc shRNA (with ML7)	D
13 kPa - Ctrl shRNA (with ML7)	B
13 kPa - Obsc shRNA (with ML7)	C
280 kPa - Ctrl shRNA (with ML7)	B
280 kPa - Obsc shRNA (with ML7)	B C

Circularity (Figure 7C)

**Games-Howell Pairwise Comparisons**<br>*Grouping Information Using 95% Confidence*<br>*Means that do not share a letter are significantly different.*	
4 kPa - Ctrl shRNA	A
4 kPa - Obsc shRNA	D E
13 kPa - Ctrl shRNA	B C
13 kPa - Obsc shRNA	E
280 kPa - Ctrl shRNA	A B
280 kPa - Obsc shRNA	C D E
4 kPa - Ctrl shRNA (with ML7)	C D E
4 kPa - Obsc shRNA (with ML7)	A B C
13 kPa - Ctrl shRNA (with ML7)	B C D
13 kPa - Obsc shRNA (with ML7)	D E
280 kPa - Ctrl shRNA (with ML7)	C D
280 kPa - Obsc shRNA (with ML7)	C D

Solidity (Figure 7D)

**Games-Howell Pairwise Comparisons**<br>*Grouping Information Using 95% Confidence*<br>*Means that do not share a letter are significantly different.*	
4 kPa - Ctrl shRNA	A
4 kPa - Obsc shRNA	C D
13 kPa - Ctrl shRNA	B
13 kPa - Obsc shRNA	D
280 kPa - Ctrl shRNA	A B
280 kPa - Obsc shRNA	B C
4 kPa - Ctrl shRNA (with ML7)	B C
4 kPa - Obsc shRNA (with ML7)	A B C
13 kPa - Ctrl shRNA (with ML7)	A B
13 kPa - Obsc shRNA (with ML7)	C D
280 kPa - Ctrl shRNA (with ML7)	B
280 kPa - Obsc shRNA (with ML7)	B C D

Grouping information is shown in all tables using a 95% confidence level. Data correspond to main text figure indicated at the top of each subtable. *Groups that do not share a letter are significantly different from each other.*

Supplementary Table S8: Following ANOVA, Games-Howell post-hoc tests were completed for pairwise comparisons between groups of data

Δ Area (Figure 7E)

**Games-Howell Pairwise Comparisons** *Grouping Information Using 95% Confidence Means that do not share a letter are significantly different.*	
4 kPa - Ctrl shRNA	D
4 kPa - Obsc shRNA	B C
13 kPa - Ctrl shRNA	B
13 kPa - Obsc shRNA	A
280 kPa - Ctrl shRNA	B C
280 kPa - Obsc shRNA	A
4 kPa - Ctrl shRNA (with ML7)	B C
4 kPa - Obsc shRNA (with ML7)	D
13 kPa - Ctrl shRNA (with ML7)	B
13 kPa - Obsc shRNA (with ML7)	C
280 kPa - Ctrl shRNA (with ML7)	B
280 kPa - Obsc shRNA (with ML7)	B C

Δ Aspect ratio (Figure 7F)

**Games-Howell Pairwise Comparisons** *Grouping Information Using 95% Confidence Means that do not share a letter are significantly different.*	
4 kPa - Ctrl shRNA	E
4 kPa - Obsc shRNA	A B
13 kPa - Ctrl shRNA	B C
13 kPa - Obsc shRNA	A
280 kPa - Ctrl shRNA	C D E
280 kPa - Obsc shRNA	A
4 kPa - Ctrl shRNA (with ML7)	A B
4 kPa - Obsc shRNA (with ML7)	E
13 kPa - Ctrl shRNA (with ML7)	E
13 kPa - Obsc shRNA (with ML7)	C D
280 kPa - Ctrl shRNA (with ML7)	D E
280 kPa - Obsc shRNA (with ML7)	C D E

Δ Circularity (Figure 7G)

**Games-Howell Pairwise Comparisons** *Grouping Information Using 95% Confidence Means that do not share a letter are significantly different.*	
4 kPa - Ctrl shRNA	E
4 kPa - Obsc shRNA	A B
13 kPa - Ctrl shRNA	B C
13 kPa - Obsc shRNA	A
280 kPa - Ctrl shRNA	C D E
280 kPa - Obsc shRNA	A
4 kPa - Ctrl shRNA (with ML7)	E
4 kPa - Obsc shRNA (with ML7)	E
13 kPa - Ctrl shRNA (with ML7)	E
13 kPa - Obsc shRNA (with ML7)	C D
280 kPa - Ctrl shRNA (with ML7)	D E
280 kPa - Obsc shRNA (with ML7)	C D

Δ Solidity (Figure 7H)

**Games-Howell Pairwise Comparisons** *Grouping Information Using 95% Confidence Means that do not share a letter are significantly different.*	
4 kPa - Ctrl shRNA	G
4 kPa - Obsc shRNA	B C
13 kPa - Ctrl shRNA	D E F
13 kPa - Obsc shRNA	A
280 kPa - Ctrl shRNA	E F G
280 kPa - Obsc shRNA	A B
4 kPa - Ctrl shRNA (with ML7)	E F
4 kPa - Obsc shRNA (with ML7)	F G
13 kPa - Ctrl shRNA (with ML7)	F
13 kPa - Obsc shRNA (with ML7)	C D
280 kPa - Ctrl shRNA (with ML7)	E F
280 kPa - Obsc shRNA (with ML7)	D E

Grouping information is shown in all tables using a 95% confidence level. Data correspond to main text figure indicated at the top of each subtable. *Groups that do not share a letter are significantly different from each other.*
